# Advancing Research Alongside Patient Partners: Next-Generation Best Practices for Effective Collaboration in Health Research

**DOI:** 10.3390/curroncol31110513

**Published:** 2024-11-07

**Authors:** Ally C. Farrell, Jessica A. Lawson, Ovarian Cancer Canada’s Patient Partners in Research Team, Alison Ross, Alicia A. Tone

**Affiliations:** Ovarian Cancer Canada, 316-4211 Yonge St., Toronto, ON M2P 2A9, Canada; afarrell@ovariancanada.org (A.C.F.); jlawson@ovariancanada.org (J.A.L.); ovcan@ovariancanada.org (Ovarian Cancer Canada’s Patient Partners in Research Team); aross@ovariancanada.org (A.R.)

**Keywords:** patient partner, patient engagement, best practices, ovarian cancer research, patient-oriented research, patient partner–researcher relationship

## Abstract

Ovarian Cancer Canada’s Patient Partners in Research (PPiR) is a national volunteer-based program that trains and connects individuals with lived ovarian cancer (OC) experience to diverse research opportunities, to maximize the clinical relevance and real-life impact of OC research in Canada. A steadily increasing demand for patient partners to be involved as research team members and decision-makers led us to co-develop with the PPiR team a series of “best practices” for researcher–patient partnerships. This framework formalizes our evolving approach to patient engagement and begins to address challenges that can arise in research settings focused on less commonly diagnosed yet significant and fatal diseases such as OC: (1) Start early. (2) Foster collaboration among the entire research team. (3) Establish expectations and communicate regularly. (4) Report impact of patient partner contributions. (5) Ensure adequate resources. While there are ongoing challenges associated with patient engagement that need to be addressed, data collected from an anonymous survey of Canadian OC researchers show a marked improvement in perceived benefits of patient engagement over time and validate the best practices presented herein. Developed in the context of OC research, these best practices can be adapted to a variety of health research settings with similar challenges.

## 1. Introduction

The role of individuals with lived experience with a particular disease in research has significantly evolved over the last 5–10 years, with an increased emphasis, implementation, and uptake of patient engagement initiatives in research settings across Australia, Europe, and North America [1,2,3]. For example, the Patient-Centered Outcomes Research Institute (PCORI) in the United States and the Strategy for Patient-Oriented Research (SPOR) in Canada have taken concrete steps to integrate patient partners into the research process [4,5]. In contrast to the term “patient”, commonly defined as an individual receiving medical care, the term “patient partner”, which will be used herein, refers to an individual with lived experience with a given disease (either firsthand or as a caregiver) who acts as a collaborator and/or decision-maker in various health research settings [6,7,8]. The scope of how patient partners contribute to health research continues to grow, but can include serving on grant review panels, research advisory councils or graduate student advisory committees, or participating as an embedded member of a research team [9]. The implementation of patient partners in health research is at the intersection of the notion “research is care” and the “nothing about us without us” initiative, established by the Anti-Apartheid Movement, that gained broader recognition through the Disability Rights Movement [10,11]. This principle emphasizes the necessity of research in the overall healthcare system and the importance of involving individuals directly affected by decisions in the decision-making process. In a health research context, this approach ensures that patients and caregivers—those most impacted by healthcare outcomes—are active participants in shaping the research agenda, methodologies, interventions, and outcomes.

To date, several guidelines on patient engagement have been released by government agencies, research institutes, and not-for-profit organizations [5,12,13]. Despite these resources, inconsistencies between disease-specific frameworks pose significant challenges. The absence of clear guidelines can lead to varied experiences for both patient partners and research teams, which may adversely impact these important collaborations [14]. Furthermore, there are very few resources detailing how best to address challenges and barriers that can arise for patient partners in research settings focused on less commonly diagnosed yet significant and fatal cancers or diseases (e.g., ovarian cancer). These issues are further compounded for members of underrepresented populations, who may face additional barriers to involvement, including (but not limited to) financial and/or time constraints and mistrust of the healthcare system, limiting their participation in shaping research that impacts their communities.

In Canada, an estimated 3000 women will be diagnosed with ovarian cancer (OC) in 2024, and 5 die from OC every day [15]. Long-term survival outcomes have not changed in over 50 years, with 56% of women diagnosed not living beyond 5 years [15]. Ovarian Cancer Canada (OCC) is the only national charity dedicated to overcoming OC (www.ovariancanada.org). Established in 1998, OCC initially focused primarily on providing information and support to individuals living with OC, with modest annual investments in research over its first 20 years. Spurred by urgent calls from patients and clinicians for new treatment options, and through collaborations with the national research community, a CAD 10 million investment by the Government of Canada to OCC in 2019 brought research to the forefront of the organization. As part of this new national research strategy (“OvCAN”), OCC established a national Patient Partners in Research (PPiR) program in 2020, to keep the voices of those with lived experience at the forefront of research.

The PPiR program is a national volunteer-based initiative that trains and connects patient partners to diverse research opportunities to enhance the clinical relevance—and thus maximize the impact—of OC research in Canada. In addition to task-specific training, training for patient partners includes the completion of the “Science of Cancer” course by the Canadian Cancer Survivor Network (https://survivornet.ca/the-science-of-cancer-online-course/, accessed on 1 September 2022) and monthly meetings where invited expert speakers deliver research presentations and/or workshops. Two OCC research staff and two patient advocates co-lead the program. Team membership includes representation of different OC types, age, sexuality, cultural backgrounds, and geography. Since its inception, the PPiR program has steadily grown in membership, scope, number and type of research engagements (from 8 in 2020 to a total of 316 by May 2024), formalization of processes, and integration into the national OC research ecosystem. An increasing demand—from funders, researchers and patients alike—for patient partners to be involved as team members and decision-makers in a variety of health research settings led us to establish a series of best practices based on our experiences and learnings over the past four years.

To our knowledge, this article is the first to outline a series of best practices for patient partner engagement that begins to address a subset of the additional and more nuanced barriers faced by those involved in OC research. The framework presented herein was co-developed with current members of the PPiR team and further refined with input from a national network of not-for-profit staff, trainees, clinicians, and scientists. While developed in the context of OC, this framework can be adapted to patient–researcher partnerships in the context of other disease sites with similar challenges.

## 2. Materials and Methods

**Figure 1:**
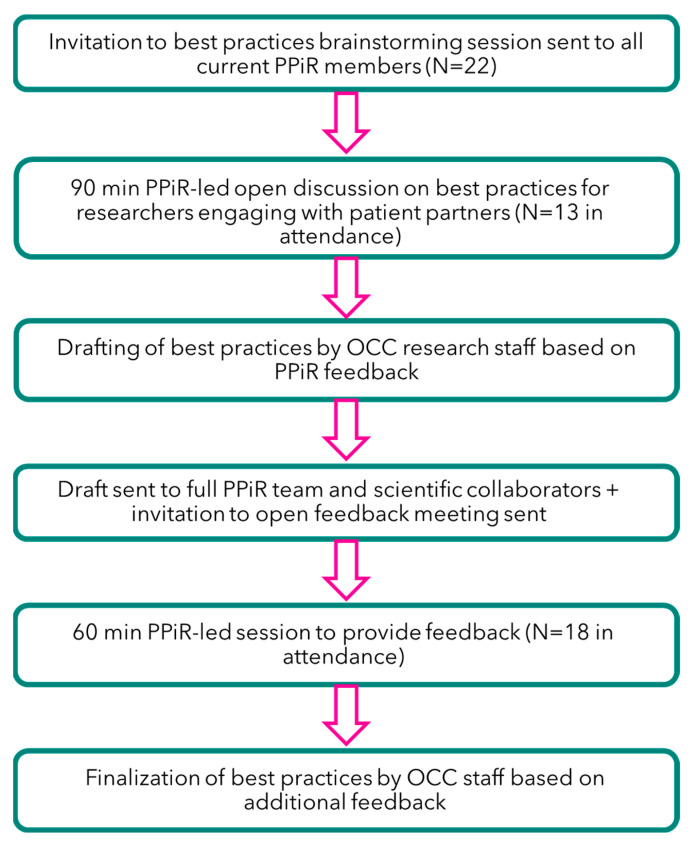
Study schema. The steps involved in co-developing the best practices framework, in collaboration with current members of the national Patient Partners in Research (PPiR) team.

**Figure 2:**
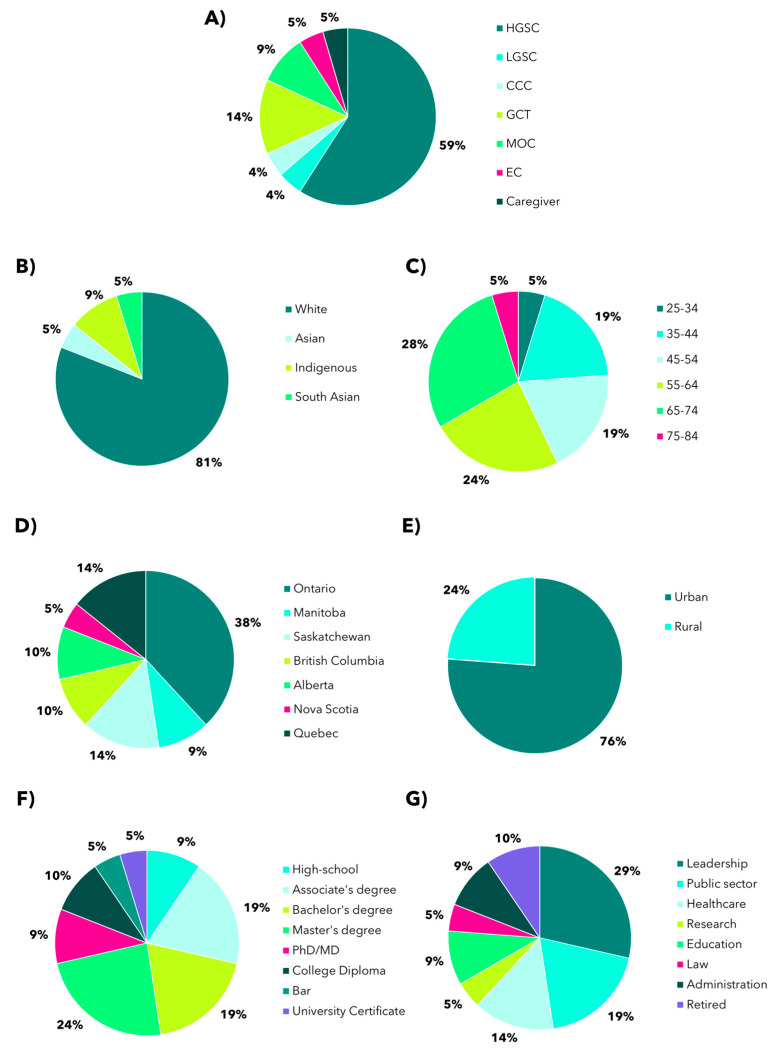
Current demographics of the PPiR team (N = 21 responses). (**A**) Proportion of PPiR (%) with high-grade serous carcinoma (HGSC), low-grade serous carcinoma (LGSC), clear cell carcinoma (CCC), granulosa cell tumor (GCT), mucinous ovarian carcinoma (MOC), and endometrioid carcinoma (EC). Breakdown of self-reported ethnicity (**B**), age range (**C**), and province of residence (**D**) of the current PPiR team. (**E**) Proportion of PPiR (%) living in urban or rural communities, where rural community is defined as having a population of less than 15,000 people. Highest level of education completed (**F**) and occupation type (**G**) of PPiR team members.

The overall study schema used to co-create a series of patient engagement best practices for our unique patient population is shown in Figure 1. The best practices development team was composed of OCC research staff and current members of the PPiR team (N = 22 members), each with diverse lived OC experiences, professional backgrounds, geographic locations, and personal circumstances. Recognizing the complexity of identities and perspectives, the team ensured multiple voices were represented throughout the creation of this framework; demographics of the current PPiR team are detailed in Figure 2. Of note, our current team includes representation of 6 types of OC, 7 provinces and 6 age categories. Our team is very well educated, with 91% of our team members having a post-secondary education. In their professional lives, many PPiR team members hold leadership roles (29%) or have worked in the public sector (19%) or healthcare/research setting (19%). Only 19% of team members self-identify as an ethnicity other than Caucasian (Indigenous, Asian, South Asian), and only 24% live in a rural community.

We acknowledge that the demographics of our current program do not fully represent the diversity of individuals with ovarian cancer (discussed further in the Discussion). To help address this, we implemented several strategies to enhance inclusivity and accessibility. Workshops were held virtually to increase participation opportunities, and participants had the option to provide real-time feedback during the workshops or later in written form. Deadlines for submitting feedback were extended upon request, ensuring flexibility for all those wishing to participate. Additionally, all members, regardless of prior collaboration burdens, were given equal opportunity to contribute to this optional project, recognizing that individuals from historically underrepresented or marginalized communities are often and may already be over-extended with requests.

All current PPiR team members were invited to participate in an open discussion-style workshop to share their views on what the “best practices” of patient engagement in health research should be, based on their own experiences. PPiR members engaged in all aspects of co-creation, from the initial brainstorming and prioritization of best practices through the development of the manuscript. Workshops were held online via Zoom Workplace (Zoom Video Communications Inc., San Jose, CA, USA) in April and September 2024, each lasting 60–90 min, with follow-up emails circulating draft strategies for real-time input and feedback. To facilitate open and honest contributions, we established ground rules for dialogue, creating a safe space where all participants felt comfortable sharing their insights. Workshops were meticulously recorded to ensure that every valuable insight was captured and could be prioritized effectively throughout the development of this framework. Insights from these discussions led to the identification and prioritization of a shortlist of best practices by OCC research staff, in collaboration with the PPiR team.

Additionally, researchers, including scientists, clinicians, and trainees, were approached for permission to include project details of previous and ongoing research collaborations with the PPiR team, to provide real-world examples of these best practices in action. Quotes were gathered through email correspondence, and relevant passages on the project being discussed were reviewed and approved by all named collaborators.

To validate the patient engagement strategy set forth by OCC, an anonymous online survey was sent in June 2024 to members of the Canadian OC research community (available online via SurveyMonkey [Team Premier version; SurveyMonkey Canada Inc., Ottawa, ON, Canada] between 7 and 21 June 2024; N = 40 responses received). This survey, circulated as part of the end of the OvCAN initiative, aimed to measure the impact of the identified research priorities included in OCC’s research strategy, including the development and implementation of the PPiR program. Participants were asked to provide insights on their experiences with the team and potential benefits of patient engagement, such as improving research design, increasing participant enrollment, creating wider impact, building rapport with patient communities, identifying research gaps and priorities, and enhancing research effectiveness. Additionally, the survey addressed potential challenges that can arise while conducting patient engagement, including cultural sensitivities, communication barriers, training needs, time constraints, and funding for logistical support and compensation. Where possible, 2024 survey responses were compared to those received from matched questions sent to the same community in 2020, prior to the launch of the PPiR program (available online via SurveyMonkey February–March 2020; N = 4 responses). As such, the findings from this survey were instrumental in validating and refining the best practices outlined herein for engaging patient partners in OC research. Categorical variables were summarized with counts and percentages while continuous variables were summarized with means, medians and ranges. The chi-squared test was used to compare proportions. Comparison of means was performed using an unpaired *t*-test.

## 3. Results

**Figure 3:**
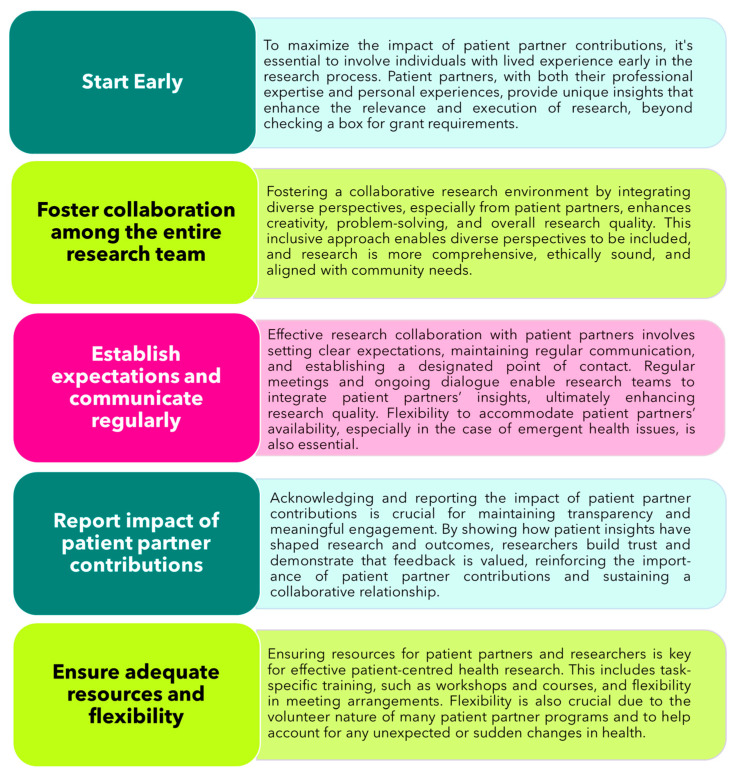
Co-developed best practices for patient engagement in OC research. This figure illustrates key best practices for effective patient engagement in ovarian cancer research. These practices, co-developed with patient partners, aim to enhance the inclusivity and effectiveness of patient-centered research initiatives.

The five best practices for patient engagement that resulted from the iterative and collaborative process outlined above are summarized in Figure 3. These seek to reinforce the growing implementation of general patient engagement initiatives in health research, while providing insights and suggestions for circumstances in which a more nuanced approach is needed. The best practices described herein aim to maximize the efforts put forth by patient partners to ensure that their perspectives remain integral to the research process. They are intended to be adaptable to similar organizations and health research settings worldwide, promoting a more consistent and impactful approach to patient engagement.

### 3.1. Start Early

To maximize the impact of patient partner contributions, it is crucial to involve individuals with lived or living experience as early as possible in the research process. This includes collaborating on research projects and programs along the translational continuum (e.g., discovery-based, pre-clinical, clinical trials, implementation research) and in the early stages of a specific project (e.g., during initial planning, rather than after funding is secured).

In addition to their pre-existing professional expertise, patient partners bring unique lived perspectives that can help guide and enhance the conceptualization and execution of research studies, making them more relevant to the affected community. It is critical to emphasize, however, that patient engagement should not be conducted to merely check a box for a grant competition or be limited to serving as an inspiration for the research being conducted (i.e., tokenism); the goal is to have patient partners serve as integral members of the research team.

An exemplary case of early patient engagement is demonstrated by Dr. Robin Urquhart, Senior Scientist at the Beatrice Hunter Research Institute and Associate Professor in the Department of Community Health and Epidemiology at Dalhousie University. As part of her cancer survivorship research program, Dr. Urquhart recently invited Yelena Aizenberg (PPiR; Ontario) and Julee Pauling (PPiR; Ontario) to collaborate on a long-term survivorship study that aims to co-design tools, resources, and/or programs to better support people living long-term with their cancer. Dr. Urquhart was proactive in integrating PPiR feedback, which strengthened the project proposal and ensured it aligned with community needs. Dr. Urquhart shared that “Incorporating lived and living experience as part of the research team is vital to ask the right questions and to develop supports and resources that meet people’s needs as they live with and beyond advanced cancer. Yelena and Julee’s expertise is a must-have—not a nice-to-have—in this research”. It is also worth noting that Yelena and Julee each have substantial experience working as a healthcare provider and in qualitative research, respectively, leaving them well positioned to leverage their professional expertise alongside their lived OC experience to provide relevant and insightful feedback for this project. Following the initial project meeting, Yelena stated that “Nowadays, cancer prevention, diagnosis and treatment get most of the attention of the research and clinical team. And for a good reason: as those are the keys to survival. But what happens after treatments and routine follow-ups stop? With more people living longer after cancer diagnosis, it is critical to understand and meet their ongoing challenges such as emotional, mental, physical challenges and help them lead healthy and fulfilling lives. As a long-term cancer survivor, patient advocate, and health care provider, I strongly believe that my lived experience can help Dr. Urquhart shape this research project in a meaningful and impactful way”.

Julee also shared that “The impacts of living beyond treatment for advanced and metastatic cancer far exceed the nature of the treatments and their side effects. They often include losses of careers, incomes, relationships, changes in family status, changes in socioeconomic status, as well as the general capacity to pursue a balanced life beyond the identity of cancer patient. These factors all cause a sudden and deeply altered sense of meaning, which other scientists and researchers are showing to also have an impact on health outcomes and overall survival. Not only are these amongst the social determinants of health, but they may very well be the criteria required for a holistic approach to patient recovery and rehabilitation. I support the goals of Dr. Urquhart’s study, and I will do what I can to bring both my experience and my skills to assist Dr. Urquhart and her team. I agree that there is an emerging and widespread need to identify the components of needed care and supports for this growing and yet underserviced population of Canadians. My hope is that my experience both as a patient in the system and in my previous academic and public service careers might together lead me to contribute meaningfully to a new paradigm of cancer support post-treatment. In particular, we aim to support interventions that help patients to not just survive longer, but to live well, with advanced disease”.

The contributions made by patient partners are not limited to research projects and can also be applied to program development in a health research setting. Dr. David Cook, a newly appointed OC Scientist at the Ottawa Hospital Research Institute and Assistant Professor in the Department of Cellular and Molecular Medicine at the University of Ottawa, actively sought feedback from the PPiR team to help design the future direction of his research. More specifically, Dr. Cook provided an overview of his training and research experience to the PPiR team before outlining some of the potential directions that his research could take as a new investigator. This was followed by an open discussion during which PPiR members were able to provide feedback and suggestions based on their lived experiences and insights gained from research projects on which they had previously collaborated.

After reflecting on his experience, Dr. Cook stated that “I recently had the opportunity to participate in a workshop with the PPiR team to help design the direction of our research moving forward. It was a humbling reminder that the voices of those with lived experience can be more illuminating than statistics in scientific papers. The session gave me clarity on under-appreciated aspects of the patient experience that I could strive to improve, including sparking my motivation to do work on the rarer subtypes of ovarian cancer, which we have since gotten two separate grants to work on! The vast majority of ovarian cancer work is on high-grade serous because of the statistics, but you know, there are still tons of women being diagnosed with these other subtypes. Plus, I do think the impact of research effort isn’t linear, and so there’s a ton to gain from even a little more effort towards these less-studied cancers. This will set the foundation for our research, and I look forward to maintaining a relationship with Patient Partners as the lab continues to grow”. Taken together, these examples highlight that by collaborating early with patient partners, researchers can develop more relevant, effective, and patient-centered research plans, leading to better policies and health interventions that truly reflect the needs and preferences of the community.

### 3.2. Foster Collaboration Among the Entire Research Team

Fostering a collaborative research environment promotes well-rounded research by combining the diverse expertise and insights of different team members, leading to more comprehensive and positive health research outcomes. By integrating diverse perspectives and fostering collaboration among the entire research team, including patient partners, researchers can enhance creativity, problem-solving, and overall research quality, ensuring that the research process is both inclusive and impactful.

This principle was clearly demonstrated in a genetic counselling project conducted at the University of Manitoba, focused on perceived OC risk and consideration of risk-reducing salpingo-oophorectomy in Hereditary Breast and Ovarian Cancer and Lynch Syndrome. The project brought together a diverse collaborative team comprising a trainee principal investigator (now genetic counsellor), Rebekah Kukurudz-Gorowski; a lab principal investigator, Dr. Mark Nachtigal; and a patient partner, Sylvia Horn (PPiR; Manitoba). More specifically, Sylvia provided the research team with critical feedback on a survey and interview guide, in addition to piloting the interview and helping to refine the statistical and thematic analyses. She also assisted in ensuring participant results were presented sensitively and clearly. Following the project, Rebekah shared that “My experience working with Sylvia has been enriching for both the study and my personal development as a researcher and healthcare professional. Her contributions from a practical aspect were important, but her experience and insight into the topic were invaluable and kept the true reason this research is important—the patients—at the heart of all stages of the research process. Working with a patient partner has opened my eyes to how every study—whether involving participants or on the bench, can benefit from patient partner collaborations”. By integrating the insights of all team members at every stage of the project, the team not only enhanced the relevance of the project but also ensured that the findings were as patient-centered as possible. Reflecting on her experience, Sylvia shared that “I had a great experience working with Rebekah. I always approach these engagements with some apprehension, mostly because I am unsure how I can make an impact. However, once we started chatting, we just naturally fed off each other. I appreciated that Rebekah kept me in the loop and continued to reach out during the project. All in all, a rewarding experience. So proud to be included”.

In addition, integrating patient partner perspectives—especially in research projects aiming to study and/or impact an underserved or marginalized community to which the research team does not belong—can also ensure that ethical and cultural considerations are thoroughly examined and addressed. As shared by Tiffany Morin (PPiR; Alberta), “As an Indigenous patient partner, ensuring that cultural practices such as ceremony is considered in research projects is important to me. Bringing in the traditional knowledge of Elders and other knowledge keepers can ensure that important aspects of collaborating with Indigenous communities is done in a good way. In meeting with researchers through OCC, I am grateful that my voice is heard and respected”.

This collaborative approach exemplifies how involving diverse perspectives can lead to more impactful, comprehensive, and culturally sensitive research strategies and outcomes in the context of OC risk management and other health research settings.

### 3.3. Establish Expectations and Communicate Regularly

The PPiR team has collectively engaged in a variety of research collaborations, including pre-clinical and clinical research projects, ranging from the identification and testing of novel therapeutic targets in OC research model systems (bench) to helping shape eligibility criteria for clinical trials (bedside). These varied experiences have highlighted the necessity of setting clear expectations and maintaining regular touchpoints between patient partners and scientific collaborators.

In our experience, setting clear expectations regarding communication and time commitments between patient partners and the research team ensures that all parties understand their roles, responsibilities, and the goals of the project, and helps to create a foundation for a purposeful and directed collaboration. For example, establishing a designated point of contact for patient partners facilitates smooth communication and ensures trust and transparency among the entire research team. Providing standard administrative documentation—such as research partner agreements and/or research engagement detail forms—have also been helpful for formalizing expectations around collaborations and ensuring that sufficient resources are available for incoming requests. For example, OCC does not currently mandate PPiR members to be compensated for their participation in research initiatives (discussed further in Section 3.5). However, it is requested that if compensation is offered that it be outlined at the time of the collaboration request. In a similar manner, regular meetings and/or communication promote ongoing dialogue and enable patient partners to openly share their insights and experiences, which enriches the research with diverse perspectives and real-world relevance. Consistent engagement with patient partners also provides an opportunity for research teams to share how patient partner feedback is being integrated and provide status updates on the collaboration where appropriate. These interactions ensure that feedback is consistently collected and integrated, not just intermittently or around deadlines. This approach also provides all parties with ongoing opportunities to provide or receive any relevant task-specific training and address potential questions or issues promptly, fostering a more responsive and effective scientific collaboration for all those involved.

In a noteworthy example of applying these principles, Dr. Laura Hopkins demonstrated exceptional practice in efficient and effective communication when collaborating with the PPiR team. Dr. Hopkins is a Gynecologic Oncologist with the Saskatchewan Cancer Agency and Professor in the Department of Oncology at the University of Saskatchewan. Dr. Hopkins led a multidisciplinary team in developing a patient decision aid form (https://decisionaid.ohri.ca/docs/das/20230804_PatientDecisionAidTool_FillableForm_Final.pdf, accessed on 1 September 2024) for OC patients with homologous recombination-proficient tumors. In forming her team, she invited a local Saskatchewan patient representative to co-create this decision aid, and an international expert in decision aid development provided structural oversight. Linda Brown (PPiR; Nova Scotia) also served as a core member of the project team prior to her death in July 2023 and contributed invaluable feedback and insights from a patient perspective on the conceptualization, design, and execution of decision aid development. This was a patient-driven project, with both patient members having a governing voice on all matters relating to chosen vocabulary and data presentation.

Once the decision aid draft was ready, Dr. Hopkins consulted with the national PPiR team to further refine the concept and its delivery. Upon making the request, Dr. Hopkins was transparent regarding the project timeline, potential workload, and the type of feedback her team was looking to receive. Thirteen PPiR members served as consultants for this project, specifically to help guide the structure, layout, and terminology of the decision aid, ensuring that it was both accessible and relevant to future users. Shannon Kadar (PPiR; Ontario) reflected on her involvement, saying, “I am honored to have been included as a patient partner in Dr. Hopkins’ patient decision aid. It was incredibly empowering to have my opinions and suggestions valued and to witness firsthand how oncology teams such as this are increasingly prioritizing patient representation. This project reaffirmed my belief that using our insights and lived experiences as front-line patients, is crucial to advancing healthcare and moving the fight against ovarian cancer forward”. Patient partners were invited to provide constructive criticisms and honest feedback, and these were reviewed to further refine the decision aid. Dr. Hopkins shared her experience, stating that “Having the patients involved from the very beginning ensures that our decision aid is credible as a patient resource—it contains the information patients want and need as opposed to the information physicians think they want and need. There is a big difference!”. This ongoing, open communication between Dr. Hopkins and the patient partners at various levels not only maximized the relevance and reliability of the decision aid but also exemplifies the meaningful contributions that patient partners can have through effective engagement and collaboration.

It is important to note that—when setting expectations and timelines for meetings or other touchpoints with patient partners in a cancer research setting—it is not uncommon for patient partners to be undergoing treatments and/or changes in health status while participating, especially during long-term projects. Medical commitments and unanticipated health setbacks may impact their availability and/or capacity for meetings at different times throughout the collaboration. This is not to suggest that assumptions should be made regarding the health status or abilities of the patient partners by scientific collaborators; rather, it emphasizes the importance of communicating with patient partners as effectively as they would with their internal research team, recognizing that accommodations and adaptations may, at times, be necessary.

### 3.4. Report Impact of Patient Partner Contributions

Acknowledging and reporting the impact of patient partner contributions is essential for fostering a continued sense of transparency, ownership, and support from the affected community. By demonstrating how patient partner insights have been integrated into patient-centered initiatives and research processes and how they have impacted results or outcomes, trainees, researchers, and clinicians can build trust and convey that the feedback is valued and taken seriously.

A compelling example of this principle occurred as part of a joint strategic competition by OCC and the Cancer Research Society (CRS) in 2021. Briefly, the aim of this national competition was to fund translational/pre-clinical research projects contributing to potential novel treatments for OC. Seven patient reviewers were selected and trained to participate and to evaluate the 39 submitted proposals based on relevance, importance and impact on the patient community, and clarity of the lay language summary. While a few PPiR members had served on grant review panels the year prior, this was the first time that they were equal voting members alongside their scientific and clinical counterparts. The impact of the patient voice was felt by both academic and patient reviewers, in real time. Dr. Jeanette Boudreau, Scientific Director of the Beatrice Hunter Cancer Research Institute and Associate Professor in the Department of Microbiology and Immunology at the University of Dalhousie, served as a scientific reviewer for this grant competition. Following the formal review process, she said “Can I just say how awesome it was to have the patients on board?! Their perspectives are so impactful—I changed my scores a number of times based on what they raised. We read about the patient experience but having them be able to share their experiences so openly … wow. You guys are doing amazing work, and I think other groups should strive to follow your lead”. Donna Pepin (PPiR; Ontario) participated as a patient reviewer, stating that “All of us on the review panel were empowered by our experience. We realized the importance of our voices as we communicated our research priorities from a perspective that only comes from a lived experience of ovarian cancer. In this journey, we are not helpless—we are helpful. This collaboration is proof of that”.

Another clear example of this was through the establishment of The Partnered Perspectives Program that was launched by OCC as part of the 11th Canadian Conference on Ovarian Cancer Research (CCOCR; May 2024) as a national networking and relationship-building opportunity for trainees in the OC research community and PPiR team members. This initiative was suggested by a PPiR member, Peggy Pickett (Ontario), inspired by a similar program organized by BioCanRX that she had previously attended. A small-scale pilot was launched in time for the conference, pairing trainees and patient partners one-on-one (nine pairs total) and having them attend a subset of the conference programming together. Following the conference, anonymous feedback from participants was collected to highlight what worked well and identify areas for future improvement. Areas for improvement included providing trainees with more notice (>1 month) before the start of the initiative, and additional opportunities for pairs to spend time together one-on-one throughout the conference. These suggestions are reflective of the quick launch and turnaround of the program and can be easily mitigated in the future. Apart from these logistical suggestions, feedback for this initiative was overwhelmingly positive from both patient partners and trainees.

Following the conference, Peggy stated that “I learned the value firsthand about the buddy system. My buddy and I sat through presentations; she helped me understand the science and after we would talk about its potential impact. Some of what she thought amazing, I thought you’ve got to be kidding me—the patient has to do what??? Some of what I thought impactful, she thought almost trivial science. It was an edifying experience for both of us; a definite win-win. I understand a lot more (about) science, and the process of bringing science from the bench to the clinic—and my buddy now believes sincerely in the value of patient partners even at the bench level of science. It is not just about knowing ‘your why’ but about getting invaluable lived experience knowledge and perspective. We built a relationship based on mutual learning and respect and are both better for it”. Her partner for the initiative, Paige Matusiak, PhD student at the Van Andel Institute (Grand Rapids, MI, USA), shared in Peggy’s sentiment, stating that “Participating in the Partnered Perspectives Program made CCOCR the best scientific conference I’ve ever attended. By providing a safe space for meaningful and vulnerable interactions with people living with OC, the initiative transformed the conference’s atmosphere into one that was deeply unifying, openly compassionate, and explicitly mindful of the real-world implications of project design and language used in research presentations. I whole-heartedly believe that the Partnered Perspectives Program can enhance the quality of life for those living with OC and those that dedicate their lives to studying it”. Another member of the PPiR team, Julie Mulligan (Ontario), shared that “The Partnered Perspectives Program is a really great way to facilitate a strong personal connection between young researchers and patients. My partner, Ramlogan, really impressed me on so many levels. In addition to being an intelligent and ambitious research scientist, he is also a wonderfully curious and gracious person to interact with socially. Instead of a brief interaction, we got to spend time chatting over the course of a couple of days and in both educational and social circumstances. I think it deepened the level of discussion and topics we covered. He was so pleasant and easy to talk to and I see big things ahead for him. I feel very privileged to have met him early in his career development. It is young researchers like Ramlogan that give so much hope for a much brighter future for OC patients”. Her partner, Ramlogan Sowamber, a PhD student at the University of British Columbia, felt similarly, stating that “Interacting with Julie was a wonderful experience that allowed me to gain insight into the value of interacting with patient partners. On our first in-person interaction, Julie and I discussed the need for alternative therapies and prevention strategies for OC, an area that requires further research. In our conversations, I quickly learned how engaged she was with research and the level of commitment she had towards the program. I believe the patient partner program sets a platform for conversations that are less likely to occur in formal environments and I am eager to discuss this experience with my colleagues and incorporate it into my research”. This initiative underscores the benefits of integrating patient perspectives into research programming and illustrates the positive impact of collaborative relationships on the next generation of health research professionals. Collectively, these examples highlight the critical role that patient partners can play in shaping research priorities and perspectives through their feedback and lived experiences in a variety of academic settings.

### 3.5. Ensure Adequate Resources and Flexibility

Ensuring adequate patient engagement resources and flexibility for patient partners as well as the research community is crucial for the success and inclusivity of health research initiatives, as they ultimately benefit all those who participate. This can include providing task-specific training and support where needed to ensure that all participants can contribute effectively and feel valued. For patient partners, this can include mentorship programs for new members, research training materials (e.g., on the ‘mechanics’ of research or basic research concepts), task-specific training (e.g., on how to participate on a grant review panel), cancer-specific workshops, or online courses, all of which can help to bridge knowledge gaps on research approaches and/or baseline knowledge of a certain topic. To ensure that all PPiR members are equipped with a general understanding of cancer biology, they are required to complete “The Science of Cancer” course administered by the Canadian Cancer Survivor Network (https://survivornet.ca/registration-for-the-science-of-cancer-online-course/, accessed on 1 September 2022) within their first year of joining, with additional tutorials provided by OCC research staff. In addition, the PPiR team meets monthly to discuss upcoming research opportunities and attend workshops and research presentations delivered by expert speakers. This enables PPiR members to establish a knowledge base of scientific principles and research processes that they can integrate alongside their lived OC experiences and professional expertise. A non-comprehensive list of resources that can be used as training materials for patient partners is included in Table 1.

For researchers, patient engagement training and equity, diversity, and inclusion (EDI) training can be an excellent starting point when preparing to engage with patient partners for a research collaboration. Adequate personnel and funding are also necessary to support these initiatives, ensuring that the engagement process is well-managed and that patient partners are properly integrated into the research team. For example, flexibility and accommodations surrounding meeting times (i.e., outside typical working hours) and means of participation (i.e., virtual or in-person) are helpful, as many patient partner programs are volunteer-based and it is not uncommon for individuals to maintain full-time employment in addition to their role as a patient partner. A limitation of this model is that it may unintentionally privilege those with the resources to be able to volunteer their time, thus excluding the perspectives of those with lived experiences who are not able to provide unpaid labor. There are some compensation frameworks that have been outlined for different types of patient engagement activities, including honoraria, travel reimbursements, and stipends [16]. In this regard, offering some form of compensation can also help to promote EDI by enabling individuals from lower socioeconomic backgrounds to contribute to research projects and helping to mitigate the costs associated with time away from work or household responsibilities. However, the topic of compensation for patient partners remains a complex and nuanced issue, as some members have expressed discomfort with the idea of being paid to share their cancer experiences, despite their roles as equal collaborators in the research [17]. This sentiment underscores the ethical and emotional dimensions of patient engagement, whereby sharing personal and often painful experiences can feel at odds with monetary compensation. The lack of a standardized approach to compensating patient partners adds to the ambiguity—there is currently no widely implemented standard for compensation in health research settings. This issue is compounded by several factors, including the absence of proper infrastructure and the lack of dedicated funding for research teams to adequately compensate patient partners in a manner that does not detract from finite operational and personnel budgets. In recognition of these systemic issues, OCC does not currently mandate that research teams offer compensation for PPiR members’ participation in research initiatives. Accordingly, the conversation around this topic is ongoing and highlights the need for more robust frameworks and resources to foster equitable patient partner compensation practices.

In a cancer research setting, it is important to be mindful of potential unexpected challenges that can occur for patient partners throughout a research collaboration (touched on in Section 3.3), including the extreme emotional and physical impact of the disease. A valuable example of how these challenges can be mitigated was observed during a collaboration between Dr. Brad Nelson, Dr. Jeanette Boudreau (mentioned above in Section 3.4), and Dr. Naoto Hirano for a novel OC immune therapy project. Dr. Nelson is a Distinguished Scientist and Director at the BC Cancer Research Institute and Professor at the University of British Columbia and the University of Victoria, and Dr. Naoto Hirano is a Senior Scientist at the Princess Margaret Cancer Centre and Professor at the University of Toronto. Briefly, their team was looking to integrate the patient perspective to aid in the conceptualization and dissemination of their research project and had also set aside the necessary resources to cover airfare for patient partners Peggy Pickett (PPiR; Ontario) and Anne Goodbody (PPiR; Ontario) to attend an in-person site visit as part of a grant application process. Importantly, Dr. Nelson and his co-applicants maintained their commitment to patient engagement when Anne was unable to attend the site visit due to unexpected changes in her health and chemotherapy regime, and instead facilitated her participation in the tour and site visit virtually. Reflecting on her experience, Anne shared that “Dr. Nelson and his team were very understanding and, with little notice, kindly enabled me to participate remotely. Peggy, who was able to attend in person, was also very gracious in keeping me in the loop. While it was truly disappointing not to be there in person, I was grateful at least to be able to participate and provide the patient perspective along with my colleague. Despite my remote participation, the team were so welcoming and inclusive to the extent that I was even included in a team photo—someone held up a laptop with me on the screen. I was truly touched by this gesture”. Overall, this last-minute accommodation enabled Anne to share insights based on her professional experience in drug development and clinical trials, alongside her lived experience with OC, which proved to be incredibly impactful for both the researchers and the members of the review committee. Following the site visit, Dr. Hirano said “I want to extend my special thanks to Peggy and Anne for their participation in the site visit. As one of the grant applicants, witnessing the interaction between reviewers, patients, and our team discussing our proposal was incredibly enlightening. This exchange added a unique and invaluable dimension to the review process, making it a standout experience among the many review panels I’ve been part of. Inspired by this, I am eager to recommend the integration of patient perspectives into the review process to CIHR and NIH next time I attend the review panel, to which I am regularly invited. Yesterday’s experience underscored the significant benefits of such engagement”. The eventual announcement by TFRI that the project had been awarded funding further showcases the impact of Peggy and Anne’s contributions.

Finally, emotional support, such as through internal or external counseling services, peer support groups, and stress management resources, is also important to help patient partners cope with survivor’s guilt and the emotional burden of their involvement, and in a similar manner can help research team members—especially trainees—to manage compassion fatigue and grief. Dr. Sarah Nersesian, a post-doctoral fellow at the Ottawa Hospital Research Institute, experienced this grief firsthand while completing her PhD program at Dalhousie University and has since highlighted the need for additional emotional support resources for both researchers and patient partners. Reflecting on her experience, Dr. Nersesian shared that “Incorporating patient partners into research not only enhances the quality and relevance of the work but also fuels motivation and passion, especially during graduate studies. These benefits are often emphasized when discussing patient partnerships. When I included a patient partner on my graduate studies advisory committee—typically comprised of academics, clinicians, and other experts—I anticipated these professional gains. However, what I didn’t expect was the deep relationship and friendship that developed with my patient partner, a bond that surpassed those with others on my committee. This relationship brought a profound sense of purpose to my research and connected me more closely with the community I aimed to impact. Tragically, in the final year of my studies, my patient partner passed away from OC. Her death left a profound mark on me, as I grappled with feelings of personal failure, knowing she didn’t get to see the project come to fruition. I often wondered, ‘If only I had worked a bit faster, maybe she would have still been there’. The grief I carried into my final year, including my advisory meetings and defense, was a poignant reminder of this loss at a time that should have been celebratory. This experience highlights the emotional complexities of partnering with patients in research. It’s essential that graduate students and researchers recognize this and ensure that emotional supports are in place for both patient partners and the researchers who work closely with them”. To the authors’ knowledge, there are currently no dedicated emotional support resources for researchers involved in patient engagement initiatives, further emphasizing the importance and urgency of addressing this unmet need. A non-comprehensive list of patient engagement resources for scientific collaborators is included in Table 2. By ensuring that these training resources and emotional supports are in place and/or are underway, research teams and patient partners can collectively foster a more inclusive (and sustainable) working environment for all those involved.

### 3.6. Best Practices Validation

**Figure 4:**
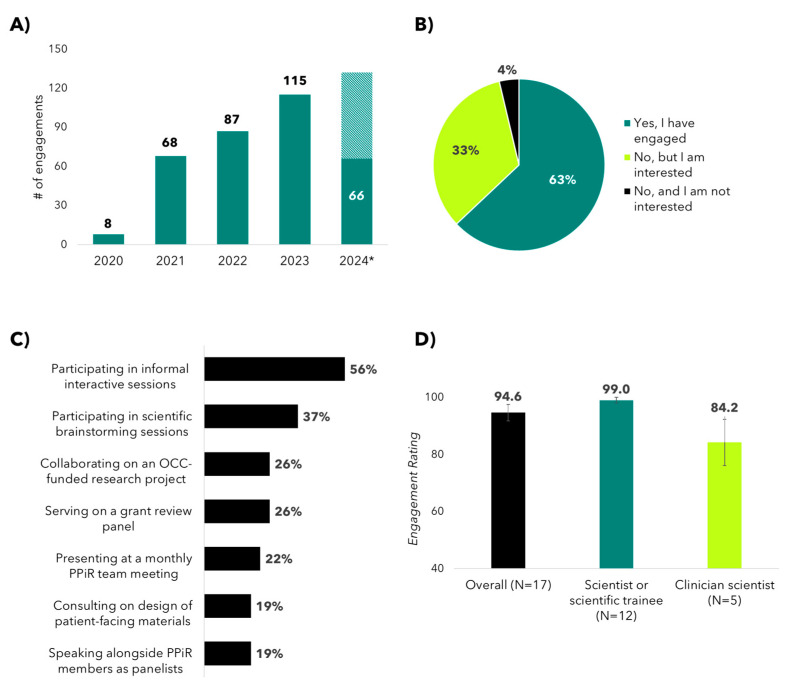
Engagement of the ovarian cancer research community with OCC’s Patient Partners in Research (PPiR) Program. (**A**) Number of unique engagements by PPiR team members by calendar year, as tracked by OCC research staff. * There were 66 engagements between January and June 2024, bringing the projected 2024 total to 132 if trends continue. (**B**) Proportion of 2024 survey respondents who have engaged, and (**C**) in what ways they have engaged, with the PPiR team. (**D**) Bar graphs showing how 2024 survey respondents rated their overall experience of engaging with OCC’s PPiR team. The trend towards an increased average score among those self-identifying as a scientist or scientific trainee is shown, compared to clinician scientists (*p* = 0.071).

Since the launch of the OvCAN initiative in 2020, the Canadian OC research community has experienced a profound shift in recognizing the essential role and benefits of meaningful patient engagement. This cultural change is evident in the steady and significant rise in the number of engagements involving members of our PPiR team, totaling more than 300 unique research engagements with collaborators across Canada to date (Figure 4). Consistent with this increased activity, survey data collected in 2024 revealed that 63% of the 40 respondents had engaged with the PPiR team and an additional 33% were interested in engaging in the future. Types of engagements included informal interactive sessions (56%); scientific brainstorming sessions (37%); serving on grant panels (26%); collaborating on an OCC-funded research project (26%); presenting at monthly PPiR team meetings (22%); speaking alongside PPiR members as panelists at OCC events or conferences (19%); and consulting on the design of patient-facing surveys or educational tools (19%). When asked to rate their overall experience when engaging with the PPiR team from 0 to 100, the average score was 94.6 and the median score was 100. Of note, there was a trend towards an increased average rating among respondents self-identifying as scientists and/or scientific trainees compared to clinician scientists (99 vs. 84.2, respectively; *p* = 0.071). The high overall satisfaction rate further supports the formalization of OCC’s patient engagement strategy as well as the effectiveness of the best practices for patient engagement that have been implemented by the PPiR team.

**Figure 5:**
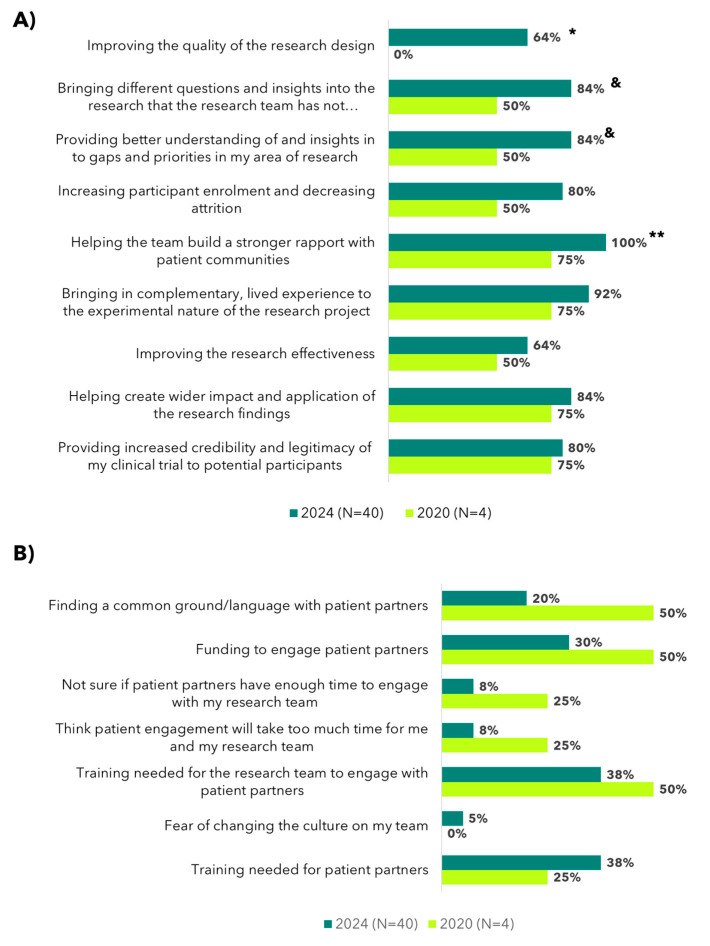
Researcher attitudes towards patient engagement in research. (**A**) The proportion of 2020 vs. 2024 survey respondents who agreed that engaging patients in their research team could benefit their research in the specific ways listed (sorted by largest to smallest increase over time). * *p* < 0.05; ^&^ *p* = 0.10; ** *p* < 0.001. (**B**) The proportion of 2020 vs. 2024 survey respondents who agreed with the specific potential challenges of patient engagement (sorted by largest to smallest decrease over time; no comparisons reached statistical significance).

In addition to increases in the number of research engagements with the PPiR team, the overall growing interest in and positive attitudes towards patient engagement in Canadian OC research is illustrated by a comparison of OvCAN survey responses (2020 vs. 2024). In 2020, a survey was circulated by OCC that included questions evaluating the attitudes towards patient engagement in OC research, of which there were only four responses (N = 4). Contrastingly, at the end of the OvCAN funding period in 2024, the number of responses had increased tenfold (N = 40). Despite a small sample size in 2020, a comparison of survey responses showed a marked improvement in the perceived benefits of patient engagement over time (Figure 5A). Of note, none of the 2020 respondents indicated that “patient engagement improved the quality of the research design”; by 2024, 64% acknowledged this benefit (*p* = 0.0144). The perception that “patient engagement increased participant enrollment and decreased attrition” rose from 50% to 80% (*p* = 0.1772). The percentage of respondents who indicated that “patient engagement provided better understanding and insights into gaps and priorities in their area of research” increased from 50% to 84% (*p* = 0.1029), which suggests that patient partners are increasingly seen as valuable contributors to identifying critical and potentially overlooked areas of research. Similarly, 84% of 2024 respondents felt that “patient partners brought different questions and insights that the research team had not considered”, up from 50% in 2020 (*p* = 0.1029), indicating a broader acceptance of the unique and clinically relevant perspectives that patient partners can offer. There was also a statistically significant increase in respondents indicating that “patient engagement helped build a stronger rapport with patient communities” (75% in 2020 vs. 100% in 2024; *p* = 0.0005).

Dr. Jim Petrik is a Tier I Canada Research Chair, Fellow in the Canadian Academy of Health Sciences, Professor at the University of Guelph in the Department of Biomedical Sciences, and an active collaborator with the PPiR team. Reflecting on the survey results, he stated that “These findings indicate that there is a growing interest in meaningful integration of patient partners in the research enterprise. To facilitate new collaborative opportunities, it will be beneficial to optimize awareness of the PPiR program within the research community across the country. Awareness programs will be essential in informing researchers who may not have attended CCOCR meetings or other OCC events that have highlighted the PPiR program. By increasing opportunities for researcher/patient partner interactions, the impact of these synergistic relationships can be maximized”.

**Figure 6:**
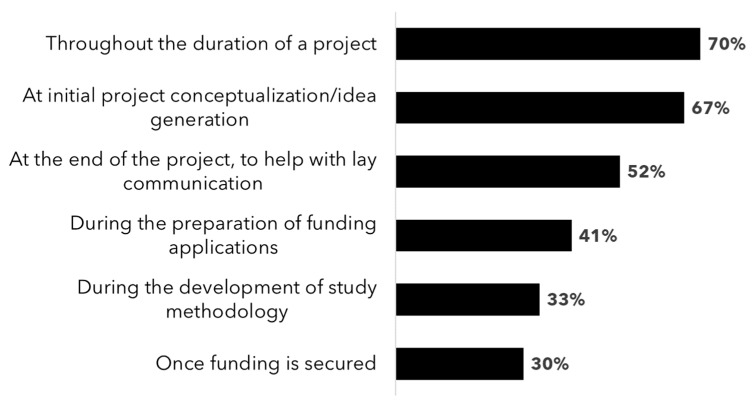
Researcher opinions on when to engage with patients, showing the proportion of 2024 respondents who answered yes to each specific scenario in response to the question “In your opinion or experience, when is the best time to engage with a patient partner when collaborating on a research project? (select all that apply)”.

The survey also evaluated perceived challenges related to patient engagement (Figure 5B), and further analysis revealed trending decreases in several key challenges related to patient engagement over time. For example, the perceived need for additional training for research teams to engage with patient partners decreased from 50% in 2020 to 38% in 2024. Similarly, concerns about funding to support logistics and compensation for patient partners dropped from 50% to 30%, and challenges in finding common ground or shared language with patient partners also decreased from 50% to 20%. Other barriers, such as “concerns regarding patient partners having sufficient time and/or availability to engage with research teams” and the perception that “engagement would take too much time from research efforts”, both fell from 25% to 8%. While none of the 2020 respondents reported concerns about changing the culture of the research team, 5% of 2024 respondents identified this as a challenge. The perceived need for patient partner training also trended upwards, from 25% in 2020 to 38% in 2024. Although perceived challenges persist and will likely need to be addressed systemically (i.e., in collaboration with funding agencies, research institutes, and patient organizations), a smaller overall percentage of survey respondents reported perceived challenges in 2024. This suggests a positive overall shift in the balance of benefits vs. challenges of patient engagement from the research community perspective. In terms of when to start the patient engagement process (Figure 6), 70% of 2024 survey respondents felt that the best time to engage with patient partners was “throughout the duration of the project”. In contrast, only 30% felt that the best time was after research funding was secured.

Dr. Barbara Vanderhyden, Chair of the OvCAN Governing Council, is a Senior Scientist at the Ottawa Hospital Research Institute and Distinguished Professor in the Department of Cellular and Molecular Medicine at the University of Ottawa. When asked to offer insights and high-level feedback on the impact of the evolving patient engagement strategy throughout the duration of the OvCAN initiative, Dr. Vanderhyden stated that “The PPiR program has achieved tremendous growth and rapid success in its recruitment, training and engagement of patient partners in a wide range of research-oriented activities. While researchers may still be learning how to accommodate the time, effort, financial and emotional toll that can come with these partnerships, there is no doubt that those who have had the opportunity to engage with patient partners have come to appreciate their contributions to research planning as well as how their different perspectives can impact research priorities. The patient partners, OCC staff and researchers have set an admirable foundation for patient engagement and are applauded for working together to make this initiative so successful”. Collectively, these data and feedback highlight areas for improvement while supporting the utility of collaborating with patient partners in OC research settings to provide important insights. Furthermore, these findings underscore the essential role that patient partners have in fostering trust and communication between Canadian researchers and the communities they serve.

## 4. Discussion

The current study outlines the next generation of best practices for patient engagement in OC research and underscores the collaborative and inclusive efforts undertaken by OCC research staff and the PPiR team. Through co-creation and continual iterative feedback, all members of the PPiR team brought a wealth of diverse professional backgrounds, lived experiences with OC, and personal circumstances, enriching this project with a multitude of perspectives. Throughout this project, we actively sought feedback from all members of the PPiR team and prioritized changes based on their input. This commitment to equity and inclusion was pivotal in shaping our approach and ensuring these perspectives were not only heard but also integrated into the final product.

It should be emphasized that the best practices presented herein are a result of more than four years of OCC staff, patient partners and researchers learning how to “do”—and more importantly, how not to do—patient engagement. From conceptualization in late 2019 and our first full team meeting in June 2020, evolution happened in real time, with both negative and positive experiences sparking the implementation of important changes for the benefit of all stakeholders. Some examples of key milestones include: (1) first patient co-lead appointed (January 2021); (2) initial conceptualization of the program vision, roles and responsibilities, and educational programming (January 2021); (3) first patient partner-trainee breakout session at virtual CCOCR (May 2021); (4) ‘buddy system’ for grant/award review process implemented (February 2022); (5) first time patient partners attended a full CCOCR scientific meeting (May 2022); (6) co-development and refinement of formal program guidelines, Research Partner agreement and PPiR volunteer agreement (August 2022–July 2023); (7) implementation of annual 1-on-1 program assessment with PPiR members (September 2022); and (8) requirement for all researchers to complete Research Engagement form prior to first collaborative meeting with patient partner/s (November 2023).

There are still many gaps that need to be addressed when it comes to PPiR program development and patient engagement initiatives overall. For example, we acknowledge that the current demographics of the PPiR team are not fully representative of the Canadian population, or the full spectrum of individuals diagnosed with OC. Briefly, in comparison to the Canadian population [18], the PPiR team has a higher proportion of individuals who self-identify as White (81% vs. 72.9%) or have a post-secondary education (91% vs. 57.5%). While the proportion living in a rural community is comparable to the Canadian population (24% vs. ~18%), we are working to grow this number further in light of the inherent challenges in implementing and accessing clinical research in this setting. These findings highlight the need for ongoing efforts to enhance diversity and representation in our work and within the PPiR program. For example, we welcome prospective PPiR members to disclose their cultural or ethnic background during recruitment. This information will aid our efforts to promote diversity and inclusivity, along with prioritizing the inclusion of individuals with different ovarian cancer types and from diverse geographical locations (e.g., rural communities). Through these workshops we identified a set of best practices that are a testament to the dedication to OC research and collaborative spirit of the PPiR team. This collaborative approach fostered a sense of collective ownership over the final version of this framework, ensuring that the outcomes are reflective of the contributions and insights of all members involved.

While the best practices outlined herein aim to address some of the systemic gaps in the field of patient engagement in health research, there are still existing challenges to keep in mind as we strive to conduct meaningful collaborations with patient partners. For example, running a volunteer-based program in Canada often poses significant challenges in achieving a representative sample of the population, leading to demographic imbalances due to selection bias. Older adults, women, and individuals with higher socioeconomic status tend to participate more frequently, while younger people, men, and those from lower socioeconomic backgrounds are generally underrepresented [19,20]. Other barriers include the time and resource commitment required for volunteering, particularly for those with demanding jobs, caregiving responsibilities, or financial constraints. The variation between available compensation frameworks for patient partners introduces another layer of complexity, as ensuring fair and equitable compensation is essential but challenging due to variations in the time commitment and burden involved in different projects [1,16,21]. For example, in a 2023 review conducted by Fox et al., 65 guidelines and policy documents on patient partner compensation were comparatively assessed [16]. Nearly all (95%) included documents recommended financial compensation for patient partners, with suggested rates between USD 12 and USD 50 per hour, varying by work type, engagement level, and time commitment. The authors concluded that there is a pressing need for unified guidance on compensation, incorporating patient partner consultation to better address population-based needs and preferences. From the perspective of research teams, budget constraints, especially in underfunded research areas like OC, also pose significant hurdles relative to other more commonly diagnosed cancer types or diseases. The lack of standardization around this factor adds to these challenges, and has left the determination of who should bear the cost of patient partner collaborations as an unresolved systemic issue that needs to be addressed [17]. Additionally, attracting volunteers from diverse cultural and linguistic backgrounds is difficult, resulting in the underrepresentation of marginalized groups. For example, Indigenous communities face significant challenges and disparities within the healthcare system, and this justified mistrust of healthcare professionals stems from a history of systemic inequities, including discriminatory practices, cultural insensitivity, and breaches of ethical standards that have adversely affected these communities. Acknowledging this historical context is essential for understanding why some individuals from these groups may approach patient partner research initiatives in healthcare with skepticism even if access is not an issue. Therefore, while the best practices outlined herein offer valuable strategies for improving patient engagement in health research, it is vital that we continue working to address persistent systemic challenges such as demographic imbalances, compensation standardization, and building trust with underrepresented groups through culturally safe approaches.

As patient partner initiatives continue to grow, it is also crucial to consider the potential emotional toll on patient organizations and research teams that work closely with patient partners, which can at times be unintentionally overlooked. Unlike healthcare professionals, it is common for staff or researchers to have less previous exposure to the emotional aspects of patient engagement, which can make the experience challenging. More specifically, in contrast to the traditional doctor–patient relationship, the patient partner–researcher relationship has evolved into a more collaborative, colleague-like dynamic, which aligns with our goals of mutual respect and shared decision-making. However, this shift can add to the complex emotions that research collaborators may experience, particularly if the patient partners they work with face a significant decline in health. This can lead to compassion fatigue, anticipatory grief, or active mourning if a patient partner’s condition worsens, or if they succumb to their disease. Despite these emotional challenges, there are no specific resources available to the research community on how to navigate these relationships. To begin addressing this gap, there is a pressing need for specific resources to support researchers in navigating these emotional challenges. For example, co-developing emotional support resources with input from grief counselors, palliative care providers, patient partners, clinicians, researchers, and trainees would serve as an excellent foundation for resources that are comprehensive, empathetic, and practical. This collaborative approach could also help to foster a sense of collegiality and support among individuals who are currently navigating these emotional challenges, enhancing the overall impact and relevance of the resources as they are developed. Additionally, research institutions should provide access to counseling services and peer support groups and expand available training on managing compassion fatigue to include researchers involved in patient engagement initiatives. Implementing policies that promote a supportive work environment, such as regular debriefing sessions and mental health days, is also crucial. By recognizing the emotional labor involved in patient engagement and providing the necessary support, we can work to maintain the well-being of scientific collaborators and others in the field to ensure that they are well-positioned to process the complex emotions that may arise when participating in patient engagement initiatives while continuing their research with resilience and empathy.

We at OCC acknowledge that there are still areas for improvement in the field of patient engagement in health research; however, the development of these best practices signifies an important advancement from earlier models that paved the way for these insights. These best practices build on four years of varied real-world collaborations with patient partners and OC researchers, addressing challenges in expanding patient engagement, especially with less commonly diagnosed diseases. Co-created with Canada’s largest group of disease-specific patient partners, the framework reflects a meaningful incorporation of diverse perspectives. Validation through the OvCAN survey results highlights a significant shift in attitudes toward patient engagement in the OC research community, the growing integration of patient partner insights into their work, and remaining areas for improvement. This underscores the model’s efficacy and the importance of maintaining an adaptive, evidence-based approach. As such, these best practices will remain relevant until they become standard practice, at which point new challenges will emerge, necessitating the development of new and improved best practices for patient engagement in health research. By continually incorporating feedback and learning from both successes and challenges, we can further improve collaboration, drive innovation in research practices, and better serve the needs of both researchers and patient partners. This iterative process ensures that our practices remain relevant and impactful, fostering meaningful and productive research partnerships.

## 5. Conclusions and Future Directions

In conclusion, the best practices for patient engagement presented herein can be applied to a variety of health research settings and are reflective of our shared commitment to advancing patient-centred research and care through inclusive and equitable practices. The continued application of OCC’s patient engagement strategy across organizational research activities beyond the now-completed OvCAN research initiative highlights its significant impact and underscores the importance of the best practices that have been developed. The marked rise in patient partner–researcher collaborations and the net positive feedback from the research community highlights a clear cultural shift towards valuing patient engagement in OC research and is consistent with the wider implementation and growing demand for patient engagement initiatives in health research settings. Furthermore, it suggests that the best practices outlined herein are effective and can be adapted for other health research settings to support patient partners facing disease-specific barriers. By fostering a culture that prioritizes meaningful patient engagement, research teams can enhance the quality, relevance, and impact of their work, ultimately leading to better outcomes for both researchers and patients across Canada.

As we look to the future, our focus will be on further advancing the PPiR program to ensure that it more accurately represents the diverse demographic of Canadians diagnosed with OC. This will involve targeted efforts to enhance participation from underrepresented groups, ensuring that research priorities and findings are more inclusive and reflective of the broader population. Additionally, formalizing a standardized compensation structure for PPiR members is a crucial step forward, as establishing fair and equitable compensation will not only address current challenges but also support sustained engagement from patient partners. This will require a careful consideration of numerous factors, including the time commitment and burden associated with different research activities, as well as budgetary constraints within the field of OC research. These efforts will lead to more representative research outcomes, strengthen collaborations with patient partners, and improve the quality and impact of OC research in Canada. Moreover, the findings from this study can be applied by research teams in other disease contexts to drive meaningful change outside of OC.

## Figures and Tables

**Table 1:** Training resources for patient partners *.

Resource	Institute	Description
The Science of Cancer (https://survivornet.ca/the-science-of-cancer-online-course/, accessed on 1 September 2022)	Canadian Cancer Survivor Network (CCSN)	Provides individuals who have lived experience with the necessary tools, skills, and background knowledge to confidently participate in a variety of research engagements.
Strategy for Patient-Oriented Research (SPOR; https://www.cihr-irsc.gc.ca/e/48413.html#a11, accessed on 1 September 2024)	Canadian Institutes of Health Research (CIHR)	An overview of Canada’s initiative for cultivating meaningful patient engagement in research. Contains definitions, current frameworks, and resources for researchers, patients, stakeholders, and the public.
CIHR Jargon Buster (https://cihr-irsc.gc.ca/e/48952.html, accessed on 1 September 2024)	CIHR	A glossary containing health research terms that have been defined in lay language.
Engagement Tool and Resource Repository (https://www.pcori.org/engagement/engagement-resources/Engagement-Tool-Resource-Repository, accessed on 1 September 2024)	Patient-Centered Outcomes Research Institute (PCORI)	PCORI is committed to advancing patient-centered, stakeholder-engaged research and the meaningful involvement of patients, caregivers, clinicians, and other healthcare stakeholders throughout the entire research process. To achieve this, they have assembled a repository of peer-reviewed engagement-related tools and resources developed and used by PCORI awardees.

* Content may also be beneficial for scientific collaborators.

**Table 2:** Training resources for scientific collaborators **.

Resource	Institute	Description
Patient Engagement Training Program (https://lms.udutu.ca/LMSPortal/Account/Logon?orgCode=IMHA, accessed on 1 September 2024)	CIHR	This program consists of modules designed to help researchers, trainees, and patient partners engage in research. It aims to enhance understanding of patient engagement and develop best practices through interactive learning exercises. The program is available in both English and French and provides a certificate upon completion.
Engaging Patients in Patient Safety—A Canadian Guide (https://www.healthcareexcellence.ca/en/resources/engaging-patients-in-patient-safety-a-canadian-guide/?gad_source=1&gclid=Cj0KCQjww5u2BhDeARIsALBuLnOxzPtSk4s8N2VmvYILGouW6nNmezAFba2H44dslnK99Vp80CfDetwaAlPyEALw_wcB, accessed on 1 September 2024)	Healthcare Excellence Canada	This guide focuses primarily on patient safety; however, many engagement practices apply to other areas, including quality, research, and education within the health research community.
The Patient Engagement Management (PEM) Suite (https://pemsuite.org)	Patient Focused Medicines Development	The PEM Suite is a hub of co-created tools, resources, and practices to help stakeholders adopt meaningful and effective patient engagement practices on a global scale.

** Content may also be beneficial for patient partners.

## Data Availability

The final coded dataset of anonymous survey responses can be made available upon request. Raw data/transcripts from workshops with PPiR membership used to develop the best practices will not be made available, to maintain privacy for the participants.
